# Anesthetic Management of a Patient With Mitochondrial Encephalomyopathy, Lactic Acidosis, and Stroke-Like Episodes Syndrome During Extensive Spinal Surgery With Both Motor Evoked Potentials and Somatosensory Evoked Potentials: A Case Report

**DOI:** 10.7759/cureus.47198

**Published:** 2023-10-17

**Authors:** Mohammad S Salehpoor, Matthew R Paluska, Ricardo Falcon, Marcus A Kuikka, Timothy R Petersen, Codruta N Soneru

**Affiliations:** 1 Department of Anesthesiology and Critical Care, University of New Mexico School of Medicine, Albuquerque, USA; 2 Department of Anesthesiology, Rocky Vista University College of Osteopathic Medicine, Englewood, USA; 3 Office of Graduate Medical Education, University of New Mexico School of Medicine, Albuquerque, USA; 4 Department of Obstetrics & Gynecology, University of New Mexico School of Medicine, Albuquerque, USA

**Keywords:** somatosensory evoked potentials, motor evoked potentials, posterior spinal fusion, anesthetic management, melas, melas syndrome

## Abstract

Mitochondrial encephalomyopathy, lactic acidosis, and stroke-like episodes (MELAS) syndrome is a complex and infrequently encountered mitochondrial cytopathy. Patients with MELAS often present with multi-systemic manifestations, making their anesthetic management particularly challenging. In this case report, we describe in detail our anesthetic approach for a 19-year-old male with confirmed MELAS linked to an m.3243A>G mutation. The patient had been diagnosed with MELAS at age 12 following a stroke-like episode and presented with progressive spinal deformities. He exhibited a 70° thoracic spine curvature and an 80° kyphosis, requiring a T1-L2 posterior spinal fusion. The surgical plan included neuromonitoring with both somatosensory and motor evoked potentials. Intravenous anesthetics such as propofol are typically preferred in this context due to their reduced interference with neuromonitoring compared to volatile anesthetics.

Anticipating a surgical duration of six to seven hours, however, we hesitated to rely on propofol for this extended period due to its potential risks of lactic acidosis in the context of MELAS. Given that propofol infusion for extended periods (>48 hours) or at high doses (≥5 mg·kg^-1^·hour^-1^) is known to induce propofol-related infusion syndrome, and coupled with our concerns about the risk of lactic acidosis in this patient, we were compelled to design an anesthetic plan that avoided propofol altogether without excessive use of volatile anesthetics. This proactive approach ensured the maintenance of consistent neuromonitoring signals and the patient’s safety, especially given his underlying mitochondrial dysfunction.

Our primary rationale in presenting this case report is to highlight the challenges posed by MELAS in the setting of extended surgery, with a focus on anesthetic considerations during neuromonitoring. For prolonged surgeries that typically rely heavily on intravenous anesthetics, which interfere less with neuromonitoring than volatile anesthetics, the use of propofol should be approached with caution in MELAS contexts due to its associated risk of lactic acidosis.

To our knowledge, this is the first case report that described the anesthetic management of a patient with MELAS undergoing a procedure of such duration, requiring both somatosensory and motor evoked potential neuromonitoring. We believe our experiences will serve as a reference for anesthesiologists and perioperative teams faced with similar challenging clinical situations.

## Introduction

Mitochondrial encephalomyopathy, lactic acidosis, and stroke-like episodes (MELAS) is a mitochondrial disorder affecting multiple systems that usually presents between two and 40 years of age. Diagnosis is based on clinical criteria and genetic testing. Our patient’s m.3243A>G pathogenic variant in the mitochondrial gene mitochondrially encoded tRNA-Leu (UUA/G) 1 is present in approximately 80% of cases [1]. MELAS is one of a spectrum of likely hundreds of mitochondrial disorders that are both genetic and environmental. Its pathophysiology is not entirely understood but likely involves dysfunction of oxidative phosphorylation within the brain, abnormalities in nitric oxide homeostasis, and decreased intramitochondrial protein synthesis [1].

Patients with MELAS require special general anesthesia considerations because of these physiologic alterations. All general anesthetics have been shown to depress mitochondrial function in some fashion. Volatile anesthetics, including desflurane, isoflurane, and sevoflurane, depress respiration, but at least one study showed that 16 patients with mitochondrial defects tolerated sevoflurane well [2].

Propofol is particularly problematic in these cases, especially with prolonged use, most likely through its inhibition of oxidative phosphorylation [3]. Patients with mitochondrial disorders are also prone to propofol-related infusion syndrome (PRIS), presenting with refractory bradycardia with metabolic acidosis, rhabdomyolysis, hyperlipidemia, and self-limiting liver injury. PRIS most often arises with prolonged infusion at high doses; the drug does appear to be safely tolerated for short-term use in some mitochondrial disorders [4].

Reported MELAS cases indicate variable responses to muscle relaxants. There have been reports of increased sensitivity to non-depolarizing muscle relaxants and resistance to cisatracurium. Because of possible comorbid kidney disease in MELAS patients, atracurium has been successfully used when relaxation was required [5]. It is essential to avoid prolonged fasting, hypoglycemia, hypothermia, acidosis, and hypovolemia to minimize additional metabolic stress.

In cases of trauma or surgery involving the spine, central nervous system ischemia or compression may occur. Intraoperative real-time evoked potential (EP) monitoring by an electrophysiologist allows the early detection of these effects. Anterior and posterior spinal cord integrity can be monitored with motor evoked potentials (MEPs) and somatosensory evoked potentials (SSEPs), respectively. With the increasing use of this neuromonitoring, the anesthesiologist must consider how anesthetics may affect EPs. Volatile anesthetics are primarily implicated in altering EPs and thus are generally avoided or used at low concentrations for most patients in whom neuromonitoring may be applied. These alterations include reduced signal amplitude and increased latency in a dose-dependent manner, so they should be kept at half minimum alveolar concentration and not varied [6]. Total intravenous anesthesia generally exhibits the most minor effect on EPs when appropriate. Opioids, midazolam, and ketamine have relatively little effect on MEPs, but propofol can depress them. If propofol is used, ketamine can counter its depressant effects. Therefore, in most cases requiring MEP monitoring, anesthesiologists may include some combination of propofol infusion, ketamine, dexmedetomidine, and an opioid.

Additionally, muscle relaxants are often avoided when MEPs must be monitored or, if used, are titrated to one or two twitches on a train of four. In addition to anesthetics, other physiologic factors can alter SSEPs and MEPs, including hypotension, hypothermia (<32°C), hypocarbia, hypoxemia, and anemia.

MELAS syndrome presents unique challenges in anesthetic management due to its associated mitochondrial defects and multi-systemic manifestations. Given propofol’s related concerns in patients with mitochondrial disorders, an anesthesia plan must be carefully formulated when neuromonitoring is to be used in these patients. This study aims to present and discuss anesthetic management challenges for a patient with MELAS syndrome requiring both MEP and SSEP neuromonitoring. To our knowledge, this is the first case report discussing anesthetic management for a MELAS patient undergoing an extended-duration spinal fusion necessitating such neuromonitoring.

This article was previously presented as a poster at the 55th annual Western Anesthesia Residents’ Conference in Portland, Oregon on April 22, 2017. Written Health Insurance Portability and Accountability Act authorization has been obtained from the patient’s legal guardian to publish this case report. This manuscript adheres to the applicable Enhancing the Quality and Transparency of Health Research Network guidelines using the Case Report checklist.

## Case presentation

A 19-year-old male with severe idiopathic adolescent kyphoscoliosis presented for a T1-L2 posterior spinal fusion to treat 70° thoracic spine curvature and 80° kyphosis, requiring neuromonitoring. His medical history was significant for premature birth, history of short stature, dysmorphic features, vitiligo, typical developmental milestones until the onset of cognitive and learning difficulties in the third grade, seizures beginning at age 13, headaches, elevated lactate during hospital admissions, and a posterior cerebellar artery infarct at age 14. Following his stroke, MELAS was confirmed with an m.3243A>G mutation. He had no prior surgical history. His family history was significant for the death of his mother due to a neuroectodermal tumor in her 20s. The patient had presented to the emergency department four months before the spinal fusion for prodromal seizure symptoms but was otherwise stable. His medications upon surgical admission included levetiracetam, lamotrigine, topiramate, clonazepam, L-arginine, coenzyme Q10, vitamin B complex, and carnitine. Laboratory testing from one month prior revealed a complete blood count significant for white blood cell count of 12.9 k/μL (4.0-11.0 k/μL) but otherwise normal, complete metabolic panel within normal limits, elevated lactate at 5.2 mmol/L (0.67-1.8 mmol/L), liver function testing significant for elevated alanine transaminase at 103 U/L (0-35 U/L), and a normal coagulation panel.

**Figure 1:**
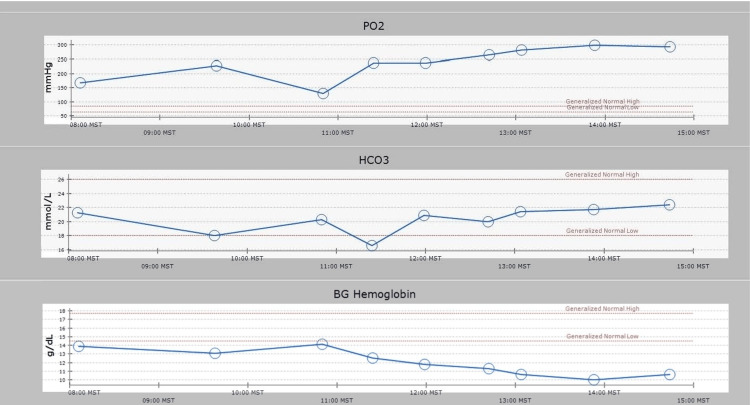
Blood gas values throughout the case. PO_2_: partial pressure of oxygen; MST: mountain standard time; HCO_3_: bicarbonate; BG: blood gas

**Figure 2:**
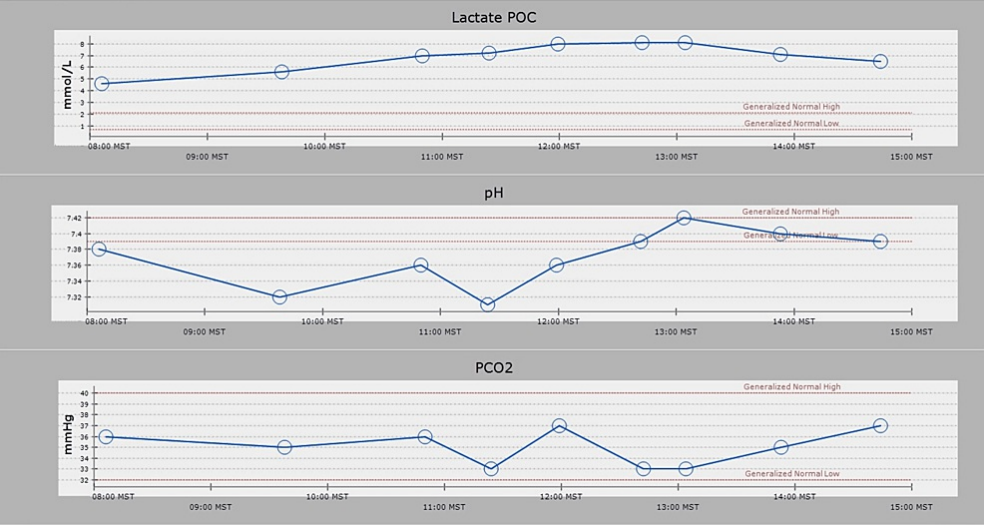
Lactate and blood gas values throughout the case. POC: point of care; MST: mountain standard time; PCO_2_: partial pressure of carbon dioxide

On the operative day, the patient’s vitals were as follows: blood pressure of 128/85 mmHg, heart rate of 137 beats/minute, respiratory rate of 20 breaths/minute, SpO_2_ of 97% on room air, temperature of 37°C, and weight of 65.5 kg. He received gabapentin 300 mg preoperatively. General anesthesia was induced with midazolam 10 mg, fentanyl 100 µg, and ketamine 30 mg, followed by rocuronium 30 mg. After general anesthesia induction, a 20-gauge radial arterial line and 8 French two-lumen internal jugular central catheters were placed. Anesthesia was maintained with <0.5 minimum alveolar concentration (MAC) of desflurane and infusions of midazolam, ketamine, remifentanil, and dexmedetomidine. After a multidisciplinary discussion between hematology/oncology, anesthesiology, and orthopedic services, we elected to use tranexamic acid despite the previous stroke to minimize bleeding. A dextrose infusion was maintained throughout the case, and the surgeon administered intrathecal morphine 0.8 mg. Mean arterial pressure was maintained above 60 mmHg. Neurophysiological monitoring included MEP, SSEP, and electroencephalography (EEG) due to concern that MEP might trigger seizures given his history. EEG demonstrated no intraoperative epileptiform activity, and SSEPs and MEPs demonstrated no deficits. Midazolam, ketamine, remifentanil, and dexmedetomidine did not negatively affect the neurophysiological monitoring quality during the procedure. Throughout the case, the serial arterial blood gas measurements remained consistently within the normal range (Figure 1). Sodium and potassium levels stayed within normal limits and demonstrated stability throughout the case. Lactate at baseline was 4.6, peaked at 8.1, and returned to 6.5 at the end of the case (Figure 2). Intraoperative blood loss was estimated at 800 mL, and the total time under anesthesia was 415 minutes. The patient received Isolyte 1,200 mL (which we chose over the lactate-containing Ringer’s lactate solution), normal saline 3,700 mL, D10 0.45 with sodium chloride 333 mL, albumin 5% 1,000 mL, and packed red blood cells 400 mL. Urine output was 2,850 mL. The patient was taken to the trauma/surgical intensive care unit, where he was extubated within 12 hours of arrival before being transferred to the medicine floor on postoperative day two.

## Discussion

MELAS syndrome is a rare syndrome in which mitochondrial protein mutations cause dysfunction in all tissues resulting in myopathy, cardiomyopathy, encephalopathy, seizure, and cerebellar ataxia (Table 1). Patients with MELAS present anesthetic dilemmas: many anesthetics may further depress mitochondrial function, sedatives and narcotics have variable dose responses, and neuromuscular blockade can be unpredictable. At our institution, a patient requiring SSEP and MEP monitoring would commonly be given a total intravenous anesthetic consisting of propofol, dexmedetomidine, and opioid infusion to avoid inhaled anesthetics’ effects on EPs, but propofol presents risks in the context of MELAS. Several case reports describe alternative anesthetic techniques for patients with MELAS. However, we could not find any existing reports discussing anesthetic management for a patient with MELAS undergoing a procedure of this duration (279 minutes of operative time and 415 minutes of anesthesia time). This appears to be the first reported case describing anesthesia care for a patient with MELAS in which SSEPs and MEPs were monitored during surgery, to our knowledge.

**Table 1:** Features of MELAS syndrome. MELAS: mitochondrial encephalomyopathy, lactic acidosis, and stroke-like episodes [7-9]

Features of MELAS syndrome
Most commonly presents between 2 and 10 years of age. Early psychomotor development is usually normal
A particular point mutation (m.3243A>G) is responsible for more than 80% of reported cases
The most common initial presenting symptoms are seizures, often with stroke-like presentation, recurrent headaches, recurrent vomiting, and hearing loss
Diagnosis is based on clinical findings, lactic acidosis, muscle biopsy (ragged red fibers), and genetic testing
Estimated prevalence of 16.3–236:100,000
The mechanism by which the mutation causes symptoms is not clear but is thought to affect mitochondrial protein synthesis and oxidative metabolism

The only other reported case of spinal surgery performed on a patient with MELAS involved muscle relaxation, which was not possible in our case because of the need to monitor MEPs. That publication described two 10-year-old sisters with MELAS who presented for posterior spinal instrumented fusion [5]. For one sister, maintenance of anesthesia included sevoflurane with boluses of atracurium and morphine. SSEPs, but not MEPs, were monitored, and the surgery lasted for less than three hours. The other sister was maintained with remifentanil and desflurane, with an operative time of 130 minutes. Another report described a safe laparoscopic appendectomy with total intravenous anesthesia using propofol and remifentanil with atracurium in a 23-year-old woman with MELAS; the procedure lasted one hour [10]. A more recent case report described success using intravenous dexmedetomidine and remifentanil for a critically ill MELAS patient undergoing a laparotomy lasting 210 minutes [11].

In one case report, a 23-year-old woman with MELAS syndrome underwent a laparoscopy-assisted appendectomy using propofol, remifentanil, and atracurium for maintenance anesthesia. The procedure was successfully performed without surgical or anesthetic complications [7]. In another case, a 53-year-old patient with MELAS underwent a gastrectomy. Propofol was utilized for anesthesia induction and maintenance. Monitoring with a bispectral index ensured values remained between 40 and 60, and there were no observed complications related to propofol administration [12]. However, we had to consider propofol’s depression of mitochondrial function and propofol infusion syndrome risk given the anticipated duration of anesthesia exposure. This possibility could not be discounted as surgery of this duration has not been described in a MELAS patient. For these reasons, we chose induction with midazolam, fentanyl, and ketamine and maintenance with desflurane at less than 0.5 MAC and infusions of midazolam, ketamine, remifentanil, and dexmedetomidine (Tables 2, 3).

**Table 2:** Effects of various anesthetic agents on MEP and SSEP latency and amplitude. Bold text indicates anesthetic agents used for maintenance. MEP: motor evoked potential; SSEP: somatosensory evoked potential; N_2_O: nitrous oxide; †: dose-dependent; *: at low doses; ‡: high dose; ↑: increase; ↓: decrease; ↔: no effect; ↘: mild decrease; ↗: mild increase; ↕: variable [13]

	MEP latency	MEP amplitude	SSEP latency	SSEP amplitude
Halothane	↑	↓	↑	↓
Isoflurane	↑	↓	↑	↓
Enflurane	↑	↓	↑	↓
Sevoflurane	↑	↓	↑	↓
Desflurane	↑	↓	↑	↓
N_2_O	↑	↓	↑	↓
Propofol	None	↓	↑	↓
Opioids	↗	↘	↔	↔
Barbituates	↑	↓	↑^†^	↔
Etomidate	↔	↕	↔	↑*
Ketamine	↗	↑* and ↓^‡^	↔	↗*
Benzodiazepines (including midazolam)	None	Sustained ↓	↑	↓
Dexmedetomidine	None	↘^‡^	↔	↔

**Table 3:** Anesthetic considerations in mitochondrial disease. [14-17]

Anesthetic considerations in mitochondrial disease	
Volatile anesthetics	Higher doses can reduce oxidative phosphorylation in mitochondria
Propofol	Inhibits multiple mitochondrial oxidative complexes. Propofol infusion syndrome can result from an indirect effect on complex II
Dexmedetomidine	No known effect on mitochondria
Opioids	Adequate analgesia minimizes oxygen demands; however, some patients with mitochondrial disease have impaired respiratory control
Benzodiazepines	Inhibit adenosine nucleotide translocase
Ketamine	Widely considered to be a safe anesthetic agent for patients with mitochondrial disorders. In vitro studies show mitochondrial depression
Neuromuscular blockers	Rhabdomyolysis with succinylcholine. Extreme sensitivity to non-depolarizers has been reported
Glucose	Fasting may result in lactic acidosis and hepatic dysfunction

With our patient’s seizure history, it was also essential to consider anesthetics’ effects on the seizure threshold. Ketamine may have both proconvulsant and anticonvulsant effects: low doses may facilitate seizures, but sedative or anesthetic doses may have anticonvulsant properties. Additionally, many documented cases of ketamine-induced seizures did not include epileptiform seizure activity on EEG, and it is known that ketamine can cause myoclonic twitches resembling seizures [18]. We also considered lidocaine infusion, as it can improve postoperative pain after complex spine surgery [19], but rejected it due to increased seizure risk with lidocaine accumulation. Despite the patient’s stroke history, we concluded after a multidisciplinary discussion that the balance of risks favored intraoperative tranexamic acid. This case presented the challenge of providing a balanced anesthetic to a patient with MELAS, where administration of propofol could cause further mitochondrial depression, and who also required neuromonitoring, where inhaled anesthetics and muscle relaxation could disrupt SSEP and MEP signals required during the surgery. This patient’s regimen provided uneventful anesthesia with appropriate neuromonitoring capability.

## Conclusions

MELAS syndrome presents unique challenges for anesthetic management. Propofol is typically the most suitable drug for anesthesia during surgeries involving intraoperative neuromonitoring because it does not interfere with neuromonitoring signals at clinically relevant doses. Its administration during total intravenous anesthesia allows for swift attainment and maintenance of the desired hypnotic effect. However, propofol presented potential risks for our patient with diagnosed MELAS, such as possible mitochondrial suppression, risk of lactic acidosis, and exacerbation of underlying metabolic disturbances.

Given these concerns, we designed a multimodal anesthetic strategy incorporating several anesthetics and avoiding both propofol and high doses of volatile anesthetics. We chose anesthesia induction with midazolam, fentanyl, and ketamine and maintenance with desflurane at 0.5 MAC and maintenance with infusions of midazolam, ketamine, remifentanil, and dexmedetomidine. This allowed us to prioritize patient safety while also preserving neuromonitoring signal integrity.

Our approach shows the importance of understanding and tailoring anesthetic regimens to the individual patient, especially in complex settings such as MELAS. Our case, to our knowledge, represents the first documented anesthetic management of a MELAS patient undergoing such prolonged surgery requiring both types of neuromonitoring.

## References

[1] El-Hattab AW, Adesina AM, Jones J, Scaglia F (2015). MELAS syndrome: clinical manifestations, pathogenesis, and treatment options. Mol Genet Metab.

[2] Morgan PG, Hoppel CL, Sedensky MM (2002). Mitochondrial defects and anesthetic sensitivity. Anesthesiology.

[3] Acco A, Comar JF, Bracht A (2004). Metabolic effects of propofol in the isolated perfused rat liver. Basic Clin Pharmacol Toxicol.

[4] Finsterer J, Frank M (2016). Propofol Is mitochondrion-toxic and may unmask a mitochondrial disorder. J Child Neurol.

[5] Loh KW, Chan CY, Chiu CK, Bin Hasan MS, Kwan MK (2016). Posterior spinal instrumented fusion for idiopathic scoliosis in patients with multisystemic neurodegenerative disorder: a report of two cases. J Orthop Surg (Hong Kong).

[6] Rosenberg AD, Marshall MH (2018). Orthopedic surgery. Basics of Anesthesia 7th Edition.

[7] El-Hattab AW, Almannai M, Scaglia F (2001). MELAS. https://www.ncbi.nlm.nih.gov/books/NBK1233/.

[8] Manwaring N, Jones MM, Wang JJ, Rochtchina E, Howard C, Mitchell P, Sue CM (2007). Population prevalence of the MELAS A3243G mutation. Mitochondrion.

[9] Majamaa K, Turkka J, Kärppä M, Winqvist S, Hassinen IE (1997). The common MELAS mutation A3243G in mitochondrial DNA among young patients with an occipital brain infarct. Neurology.

[10] Park JS, Baek CW, Kang H, Cha SM, Park JW, Jung YH, Woo YC (2010). Total intravenous anesthesia with propofol and remifentanil in a patient with MELAS syndrome -a case report-. Korean J Anesthesiol.

[11] Kim SH, Park SY, Jung KT (2019). Dexmedetomidine as a non-triggering anesthetic agent in a patient with MELAS syndrome and systemic sepsis - a case report. Anesth Pain Med (Seoul).

[12] Sasano N, Fujita Y, So M, Sobue K, Sasano H, Katsuya H (2007). Anesthetic management of a patient with mitochondrial myopathy, encephalopathy, lactic acidosis, and stroke-like episodes (MELAS) during laparotomy. J Anesth.

[13] Shils JL, Sloan TB (2015). Intraoperative neuromonitoring. Int Anesthesiol Clin.

[14] Heard J, Martin D, Tobias JD, Schloss B (2013). Dexmedetomidine and ketamine sedation for a patient with presumed mitochondrial disease and malignant hyperthermia. Anaesth Pain Intensive Care.

[15] Parikh S, Goldstein A, Karaa A (2017). Patient care standards for primary mitochondrial disease: a consensus statement from the Mitochondrial Medicine Society. Genet Med.

[16] Wallace JJ, Perndt H, Skinner M (1998). Anaesthesia and mitochondrial disease. Paediatr Anaesth.

[17] Shipton EA, Prosser DO (2004). Mitochondrial myopathies and anaesthesia. Eur J Anaesthesiol.

[18] Perks A, Cheema S, Mohanraj R (2012). Anaesthesia and epilepsy. Br J Anaesth.

[19] Farag E, Ghobrial M, Sessler DI (2013). Effect of perioperative intravenous lidocaine administration on pain, opioid consumption, and quality of life after complex spine surgery. Anesthesiology.

